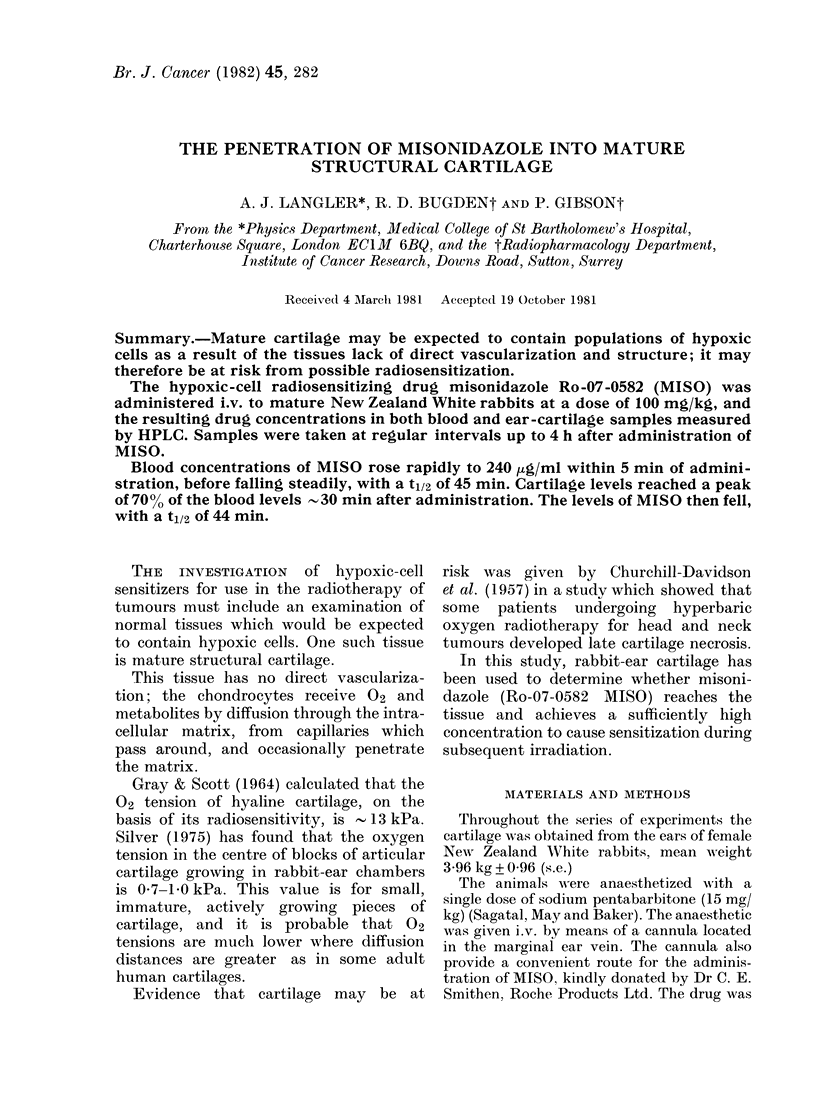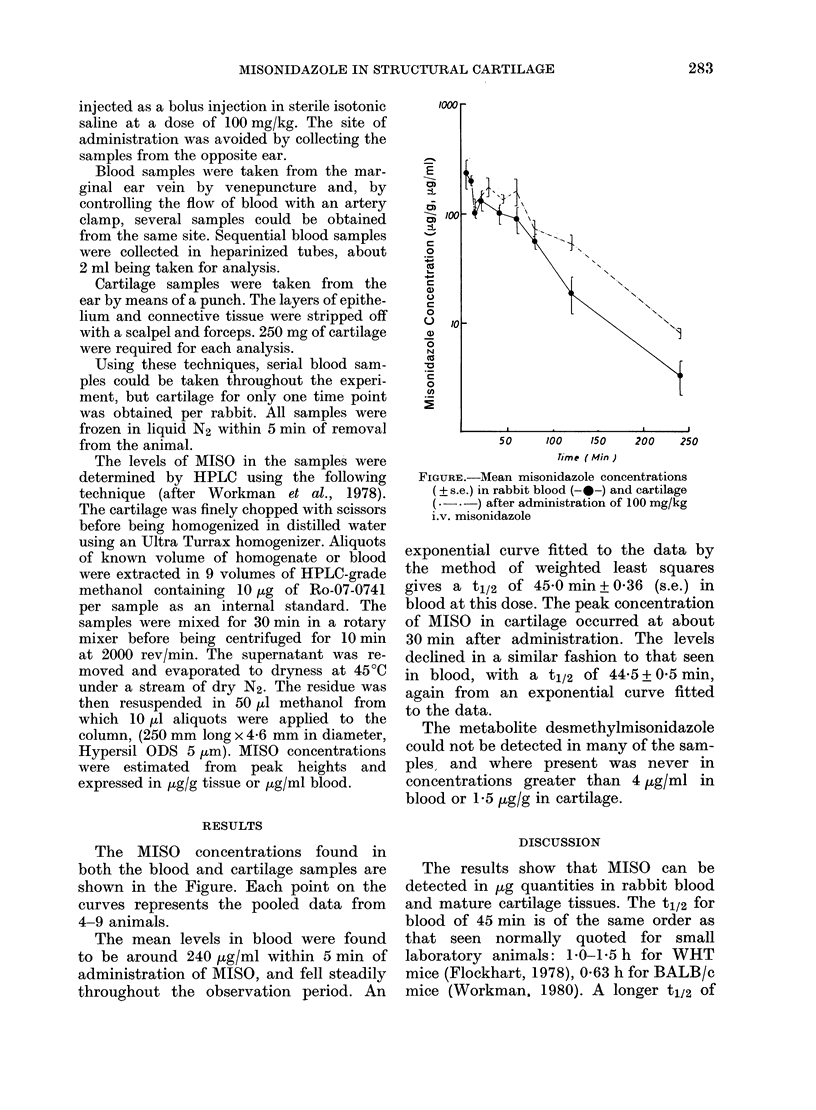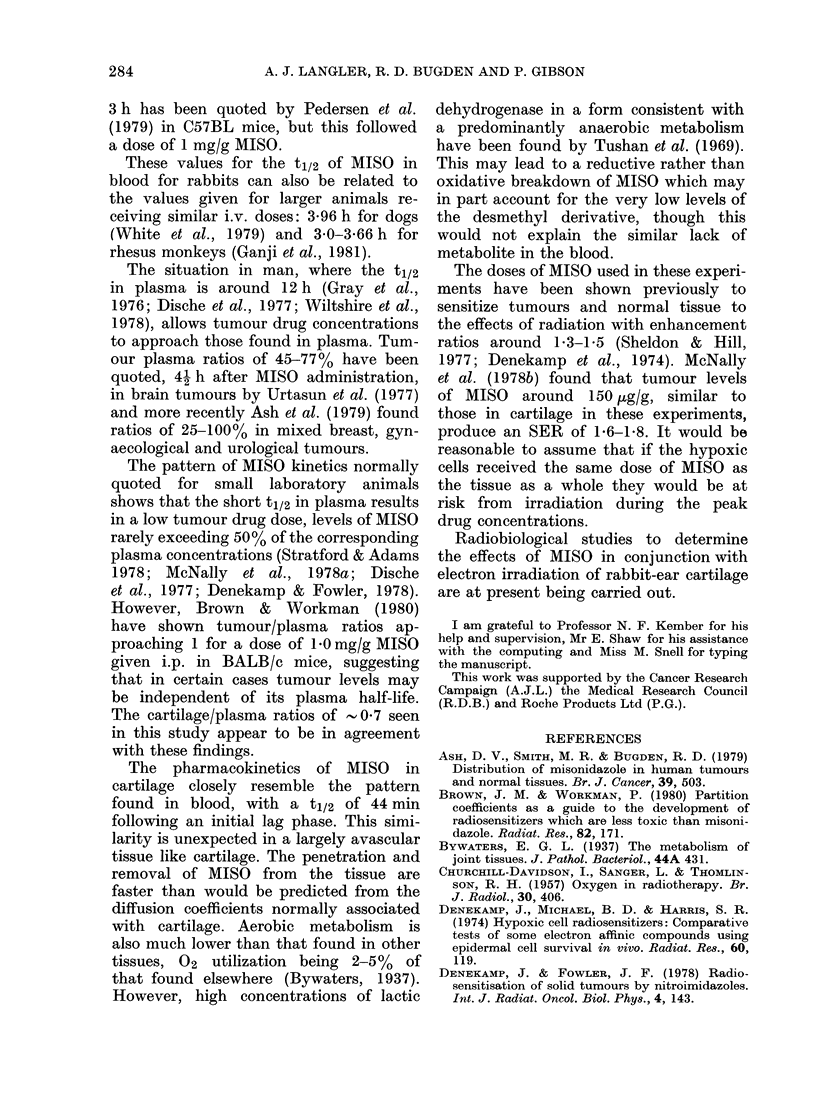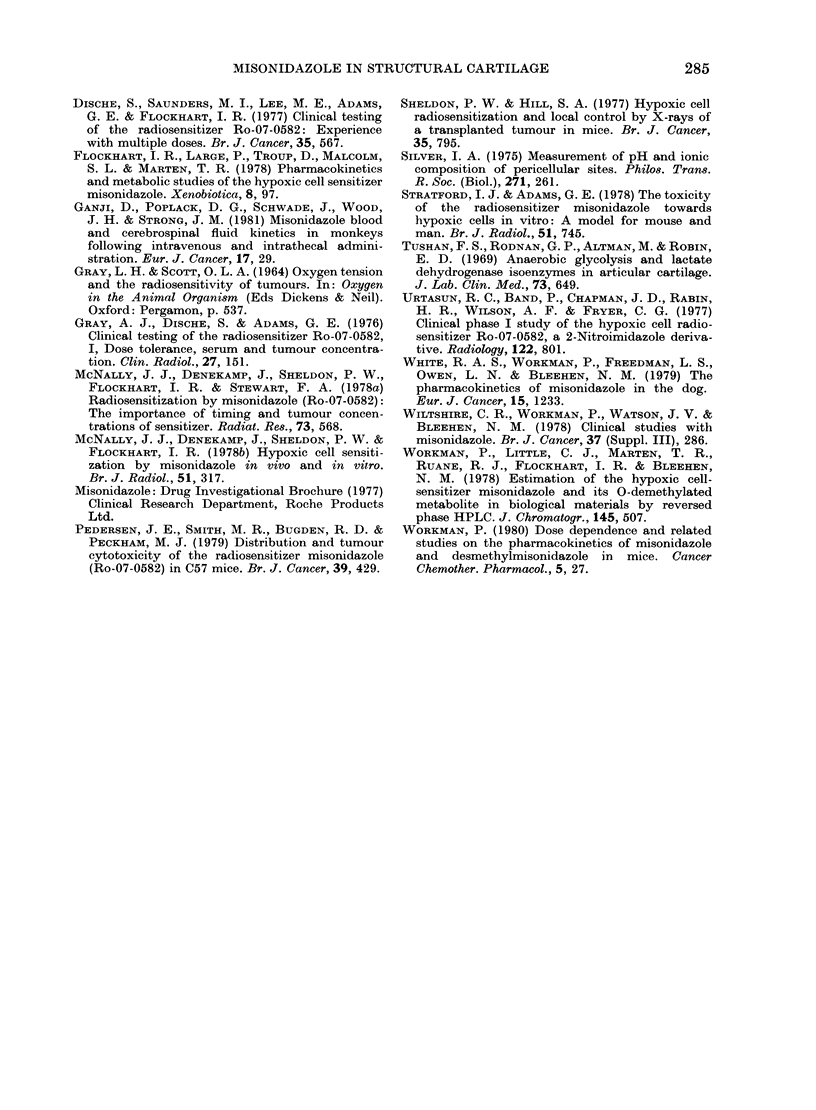# The penetration of misonidazole into a mature structural cartilage.

**DOI:** 10.1038/bjc.1982.44

**Published:** 1982-02

**Authors:** A. J. Langler, R. D. Bugden, P. Gibson

## Abstract

Mature cartilage may be expected to contain populations of hypoxic cells as a result of the tissues lack of direct vascularization and structure; it may therefore be at risk from possible radiosensitization. The hypoxic-cell radiosensitizing drug misonidazole Ro-07-0582 (MISO) was administered i.v. to mature New Zealand White rabbits at a dose of 100 mg/kg, and the resulting drug concentrations in both blood and ear-cartilage samples measured by HPLC. Samples were taken at regular intervals up to 4 h after administration of MISO. Blood concentrations of MISO rose rapidly to 240 microgram/ml within 5 min of administration, before falling steadily, with a t1/2 of 45 min. Cartilage levels reached a peak of 70% of the blood levels approximately 30 min after administration. The levels of MISO then fell, with a t1/2 of 44 min.


*Br. J. Cancer* (1982) **45**, 282

### THE PENETRATION OF MISONIDAZOLE INTO MATURE STRUCTURAL CARTILAGE

A. J. LANGLER*, R. D. BUGDEN† AND P. GIBSON†

*From the *Physics Department, Medical College of St Bartholomew's Hospital, Charterhouse Square, London EC1M 6BQ, and the †Radiopharmacology Department, Institute of Cancer Research, Downs Road, Sutton, Surrey*

Received 4 March 1981 Accepted 19 October 1981

**Summary.—Mature cartilage may be expected to contain populations of hypoxic cells as a result of the tissues lack of direct vascularization and structure; it may therefore be at risk from possible radiosensitization.**

**The hypoxic-cell radiosensitizing drug misonidazole Ro-07-0582 (MISO) was administered i.v. to mature New Zealand White rabbits at a dose of 100 mg/kg, and the resulting drug concentrations in both blood and ear-cartilage samples measured by HPLC. Samples were taken at regular intervals up to 4 h after administration of MISO.**

**Blood concentrations of MISO rose rapidly to 240 $\mu$g/ml within 5 min of administration, before falling steadily, with a $t_{1/2}$ of 45 min. Cartilage levels reached a peak of 70% of the blood levels ~30 min after administration. The levels of MISO then fell, with a $t_{1/2}$ of 44 min.**

THE INVESTIGATION of hypoxic-cell sensitizers for use in the radiotherapy of tumours must include an examination of normal tissues which would be expected to contain hypoxic cells. One such tissue is mature structural cartilage.

This tissue has no direct vascularization; the chondrocytes receive $O_2$ and metabolites by diffusion through the intracellular matrix, from capillaries which pass around, and occasionally penetrate the matrix.

Gray & Scott (1964) calculated that the $O_2$ tension of hyaline cartilage, on the basis of its radiosensitivity, is ~13 kPa. Silver (1975) has found that the oxygen tension in the centre of blocks of articular cartilage growing in rabbit-ear chambers is 0·7–1·0 kPa. This value is for small, immature, actively growing pieces of cartilage, and it is probable that $O_2$ tensions are much lower where diffusion distances are greater as in some adult human cartilages.

Evidence that cartilage may be at risk was given by Churchill-Davidson *et al.* (1957) in a study which showed that some patients undergoing hyperbaric oxygen radiotherapy for head and neck tumours developed late cartilage necrosis.

In this study, rabbit-ear cartilage has been used to determine whether misonidazole (Ro-07-0582 MISO) reaches the tissue and achieves a sufficiently high concentration to cause sensitization during subsequent irradiation.

### MATERIALS AND METHODS

Throughout the series of experiments the cartilage was obtained from the ears of female New Zealand White rabbits, mean weight 3·96 kg ± 0·96 (s.e.)

The animals were anaesthetized with a single dose of sodium pentabarbitone (15 mg/kg) (Sagatal, May and Baker). The anaesthetic was given i.v. by means of a cannula located in the marginal ear vein. The cannula also provide a convenient route for the administration of MISO, kindly donated by Dr C. E. Smithen, Roche Products Ltd. The drug was injected as a bolus injection in sterile isotonic saline at a dose of 100 mg/kg. The site of administration was avoided by collecting the samples from the opposite ear.

Blood samples were taken from the marginal ear vein by venepuncture and, by controlling the flow of blood with an artery clamp, several samples could be obtained from the same site. Sequential blood samples were collected in heparinized tubes, about 2 ml being taken for analysis.

Cartilage samples were taken from the ear by means of a punch. The layers of epithelium and connective tissue were stripped off with a scalpel and forceps. 250 mg of cartilage were required for each analysis.

Using these techniques, serial blood samples could be taken throughout the experiment, but cartilage for only one time point was obtained per rabbit. All samples were frozen in liquid $N_2$ within 5 min of removal from the animal.

The levels of MISO in the samples were determined by HPLC using the following technique (after Workman *et al.*, 1978). The cartilage was finely chopped with scissors before being homogenized in distilled water using an Ultra Turrax homogenizer. Aliquots of known volume of homogenate or blood were extracted in 9 volumes of HPLC-grade methanol containing 10 μg of Ro-07-0741 per sample as an internal standard. The samples were mixed for 30 min in a rotary mixer before being centrifuged for 10 min at 2000 rev/min. The supernatant was removed and evaporated to dryness at 45°C under a stream of dry $N_2$. The residue was then resuspended in 50 μl methanol from which 10 μl aliquots were applied to the column, (250 mm long × 4·6 mm in diameter, Hypersil ODS 5 μm). MISO concentrations were estimated from peak heights and expressed in μg/g tissue or μg/ml blood.

### RESULTS

The MISO concentrations found in both the blood and cartilage samples are shown in the Figure. Each point on the curves represents the pooled data from 4–9 animals.

The mean levels in blood were found to be around 240 μg/ml within 5 min of administration of MISO, and fell steadily throughout the observation period. An

FIGURE.—Mean misonidazole concentrations (± s.e.) in rabbit blood (–●–) and cartilage (·—·—) after administration of 100 mg/kg i.v. misonidazole exponential curve fitted to the data by the method of weighted least squares gives a $t_{1/2}$ of 45·0 min ± 0·36 (s.e.) in blood at this dose. The peak concentration of MISO in cartilage occurred at about 30 min after administration. The levels declined in a similar fashion to that seen in blood, with a $t_{1/2}$ of 44·5 ± 0·5 min, again from an exponential curve fitted to the data.

The metabolite desmethylmisonidazole could not be detected in many of the samples, and where present was never in concentrations greater than 4 μg/ml in blood or 1·5 μg/g in cartilage.

### DISCUSSION

The results show that MISO can be detected in μg quantities in rabbit blood and mature cartilage tissues. The $t_{1/2}$ for blood of 45 min is of the same order as that seen normally quoted for small laboratory animals: 1·0–1·5 h for WHT mice (Flockhart, 1978), 0·63 h for BALB/c mice (Workman, 1980). A longer $t_{1/2}$ of

3 h has been quoted by Pedersen *et al.* (1979) in C57BL mice, but this followed a dose of 1 mg/g MISO.

These values for the $t_{1/2}$ of MISO in blood for rabbits can also be related to the values given for larger animals receiving similar i.v. doses: 3·96 h for dogs (White *et al.*, 1979) and 3·0–3·66 h for rhesus monkeys (Ganji *et al.*, 1981).

The situation in man, where the $t_{1/2}$ in plasma is around 12 h (Gray *et al.*, 1976; Dische *et al.*, 1977; Wiltshire *et al.*, 1978), allows tumour drug concentrations to approach those found in plasma. Tumour plasma ratios of 45–77% have been quoted, 4½ h after MISO administration, in brain tumours by Urtasun *et al.* (1977) and more recently Ash *et al.* (1979) found ratios of 25–100% in mixed breast, gynaecological and urological tumours.

The pattern of MISO kinetics normally quoted for small laboratory animals shows that the short $t_{1/2}$ in plasma results in a low tumour drug dose, levels of MISO rarely exceeding 50% of the corresponding plasma concentrations (Stratford & Adams 1978; McNally *et al.*, 1978*a*; Dische *et al.*, 1977; Denekamp & Fowler, 1978). However, Brown & Workman (1980) have shown tumour/plasma ratios approaching 1 for a dose of 1·0 mg/g MISO given i.p. in BALB/c mice, suggesting that in certain cases tumour levels may be independent of its plasma half-life. The cartilage/plasma ratios of ~0·7 seen in this study appear to be in agreement with these findings.

The pharmacokinetics of MISO in cartilage closely resemble the pattern found in blood, with a $t_{1/2}$ of 44 min following an initial lag phase. This similarity is unexpected in a largely avascular tissue like cartilage. The penetration and removal of MISO from the tissue are faster than would be predicted from the diffusion coefficients normally associated with cartilage. Aerobic metabolism is also much lower than that found in other tissues, $O_2$ utilization being 2–5% of that found elsewhere (Bywaters, 1937). However, high concentrations of lactic dehydrogenase in a form consistent with a predominantly anaerobic metabolism have been found by Tushan *et al.* (1969). This may lead to a reductive rather than oxidative breakdown of MISO which may in part account for the very low levels of the desmethyl derivative, though this would not explain the similar lack of metabolite in the blood.

The doses of MISO used in these experiments have been shown previously to sensitize tumours and normal tissue to the effects of radiation with enhancement ratios around 1·3–1·5 (Sheldon & Hill, 1977; Denekamp *et al.*, 1974). McNally *et al.* (1978*b*) found that tumour levels of MISO around 150 μg/g, similar to those in cartilage in these experiments, produce an SER of 1·6–1·8. It would be reasonable to assume that if the hypoxic cells received the same dose of MISO as the tissue as a whole they would be at risk from irradiation during the peak drug concentrations.

Radiobiological studies to determine the effects of MISO in conjunction with electron irradiation of rabbit-ear cartilage are at present being carried out.

I am grateful to Professor N. F. Kember for his help and supervision, Mr E. Shaw for his assistance with the computing and Miss M. Snell for typing the manuscript.

This work was supported by the Cancer Research Campaign (A.J.L.) the Medical Research Council (R.D.B.) and Roche Products Ltd (P.G.).

### REFERENCES

Ash, D. V., Smith, M. R. & Bugden, R. D. (1979) Distribution of misonidazole in human tumours and normal tissues. *Br. J. Cancer*, **39**, 503.

Brown, J. M. & Workman, P. (1980) Partition coefficients as a guide to the development of radiosensitizers which are less toxic than misonidazole. *Radiat. Res.*, **82**, 171.

Bywaters, E. G. L. (1937) The metabolism of joint tissues. *J. Pathol. Bacteriol.*, **44A** 431.

Churchill-Davidson, I., Sanger, L. & Thomlinson, R. H. (1957) Oxygen in radiotherapy. *Br. J. Radiol.*, **30**, 406.

Denekamp, J., Michael, B. D. & Harris, S. R. (1974) Hypoxic cell radiosensitizers: Comparative tests of some electron affinic compounds using epidermal cell survival *in vivo*. *Radiat. Res.*, **60**, 119.

Denekamp, J. & Fowler, J. F. (1978) Radiosensitisation of solid tumours by nitroimidazoles. *Int. J. Radiat. Oncol. Biol. Phys.*, **4**, 143.

DISCHE, S., SAUNDERS, M. I., LEE, M. E., ADAMS, G. E. & FLOCKHART, I. R. (1977) Clinical testing of the radiosensitizer Ro-07-0582: Experience with multiple doses. *Br. J. Cancer*, **35**, 567.

FLOCKHART, I. R., LARGE, P., TROUP, D., MALCOLM, S. L. & MARTEN, T. R. (1978) Pharmacokinetics and metabolic studies of the hypoxic cell sensitizer misonidazole. *Xenobiotica*, **8**, 97.

GANJI, D., POPLACK, D. G., SCHWADE, J., WOOD, J. H. & STRONG, J. M. (1981) Misonidazole blood and cerebrospinal fluid kinetics in monkeys following intravenous and intrathecal administration. *Eur. J. Cancer*, **17**, 29.

GRAY, L. H. & SCOTT, O. L. A. (1964) Oxygen tension and the radiosensitivity of tumours. In: *Oxygen in the Animal Organism* (Eds Dickens & Neil). Oxford: Pergamon, p. 537.

GRAY, A. J., DISCHE, S. & ADAMS, G. E. (1976) Clinical testing of the radiosensitizer Ro-07-0582, I, Dose tolerance, serum and tumour concentration. *Clin. Radiol.*, **27**, 151.

MCNALLY, J. J., DENEKAMP, J., SHELDON, P. W., FLOCKHART, I. R. & STEWART, F. A. (1978*a*) Radiosensitization by misonidazole (Ro-07-0582): The importance of timing and tumour concentrations of sensitizer. *Radiat. Res.*, **73**, 568.

MCNALLY, J. J., DENEKAMP, J., SHELDON, P. W. & FLOCKHART, I. R. (1978*b*) Hypoxic cell sensitization by misonidazole *in vivo* and *in vitro*. *Br. J. Radiol.*, **51**, 317.

Misonidazole: Drug Investigational Brochure (1977) Clinical Research Department, Roche Products Ltd.

PEDERSEN, J. E., SMITH, M. R., BUGDEN, R. D. & PECKHAM, M. J. (1979) Distribution and tumour cytotoxicity of the radiosensitizer misonidazole (Ro-07-0582) in C57 mice. *Br. J. Cancer*, **39**, 429.

SHELDON, P. W. & HILL, S. A. (1977) Hypoxic cell radiosensitization and local control by X-rays of a transplanted tumour in mice. *Br. J. Cancer*, **35**, 795.

SILVER, I. A. (1975) Measurement of pH and ionic composition of pericellular sites. *Philos. Trans. R. Soc.* (Biol.), **271**, 261.

STRATFORD, I. J. & ADAMS, G. E. (1978) The toxicity of the radiosensitizer misonidazole towards hypoxic cells in vitro: A model for mouse and man. *Br. J. Radiol.*, **51**, 745.

TUSHAN, F. S., RODNAN, G. P., ALTMAN, M. & ROBIN, E. D. (1969) Anaerobic glycolysis and lactate dehydrogenase isoenzymes in articular cartilage. *J. Lab. Clin. Med.*, **73**, 649.

URTASUN, R. C., BAND, P., CHAPMAN, J. D., RABIN, H. R., WILSON, A. F. & FRYER, C. G. (1977) Clinical phase I study of the hypoxic cell radiosensitizer Ro-07-0582, a 2-Nitroimidazole derivative. *Radiology*, **122**, 801.

WHITE, R. A. S., WORKMAN, P., FREEDMAN, L. S., OWEN, L. N. & BLEEHEN, N. M. (1979) The pharmacokinetics of misonidazole in the dog. *Eur. J. Cancer*, **15**, 1233.

WILTSHIRE, C. R., WORKMAN, P., WATSON, J. V. & BLEEHEN, N. M. (1978) Clinical studies with misonidazole. *Br. J. Cancer*, **37** (Suppl. III), 286.

WORKMAN, P., LITTLE, C. J., MARTEN, T. R., RUANE, R. J., FLOCKHART, I. R. & BLEEHEN, N. M. (1978) Estimation of the hypoxic cell-sensitizer misonidazole and its O-demethylated metabolite in biological materials by reversed phase HPLC. *J. Chromatogr.*, **145**, 507.

WORKMAN, P. (1980) Dose dependence and related studies on the pharmacokinetics of misonidazole and desmethylmisonidazole in mice. *Cancer Chemother. Pharmacol.*, **5**, 27.